# Neurodevelopmental Outcomes of Young Children Born to HIV-Infected Mothers: A Pilot Study

**DOI:** 10.3389/fped.2021.697091

**Published:** 2021-10-21

**Authors:** Megan S. McHenry, Eren Oyungu, Ziyi Yang, Ananda R. Ombitsa, Cleophas Cherop, Rachel C. Vreeman

**Affiliations:** ^1^Indiana University School of Medicine, Indianapolis, IN, United States; ^2^Academic Model Providing Access to Healthcare, Eldoret, Kenya; ^3^Department of Child Health, College of Health Sciences, Moi University, Eldoret, Kenya; ^4^Department of Global Health, Icahn School of Medicine at Mount Sinai, New York, NY, United States; ^5^Arnhold Institute for Global Health, New York, NY, United States

**Keywords:** child development, HIV, HIV exposure, Kenya, HIV-infected, neurodevelopment

## Abstract

**Introduction:** Over 15 million children who were exposed to HIV perinatally but uninfected (HEU) are alive globally, and they are faced with multiple risk factors for poor neurodevelopment. While children who are HIV-infected (HIV+) appear to have worse neurodevelopmental scores compared to children unexposed and uninfected with HIV (HUU), the evidence is mixed in children who are HEU. This small descriptive pilot study aimed to compare neurodevelopmental scores of children who are HIV+, HEU, and HUU in Kenya.

**Methods:** This cross-sectional pilot study included children ages 18–36 months who were HIV+, HEU, or HUU. Neurodevelopment was assessed, along with sociodemographic, lab, and growth data. Statistical analysis included descriptive statistics, one-way ANOVA, chi-squared, and adjusted linear regression models.

**Results:** One hundred seventy two were included (*n* = 24 HIV+; *n* = 74 HEU; *n* = 74 HUU). Mothers of children who were HEU experienced more depressive symptoms (*p* < 0.001). The only neurodevelopmental differences were found among groups was that children who were HIV+ had higher receptive language scores (*p* = 0.007). Lower height-for-age z-scores and being left home alone were associated with worse neurodevelopmental scores.

**Conclusions:** Being stunted, left completely alone for at least an hour within the last week, and having higher sociodemographic status were associated with worse neurodevelopmental scores. The higher levels of depressive symptoms within mothers of children who are HEU warrants further investigation.

## Introduction

Children born to mothers living with HIV are increasingly able to survive but are at still at higher risk of morbidity and mortality compared to their peers who are unexposed (1). In some countries, >15% of all children are born to mothers living with HIV (2), accounting for many of the estimated 14.8 million children under 15 years of age who are HIV-exposed but uninfected (HEU) worldwide (2). Exposure to HIV or antiretroviral therapy (ART) may impact neurodevelopmental outcomes among children who are HEU. Worse neurodevelopmental scores, particularly in the domains of receptive and expressive language, have been reported in young children who are HEU and exposed to ART compared to HUU (3–6). However, other studies have found no neurodevelopmental differences between children who are HEU and HUU (7, 8). With the current inconclusive evidence, further investigation is required to delineate the neurodevelopmental impact of HIV exposure (1).

This small descriptive cross-sectional pilot study aimed to use a culturally adapted Bayley Scales of Infant and Toddler Development, 3rd Edition (Bayley-3) to measure and compare cognitive, language, and motor domains within Kenyan children ages 18–36 months who are HIV+, HEU, or HUU.

## Methods

### Study Design and Population

This was a descriptive cross-sectional pilot study assessing neurodevelopment among young children in Kenya as part of the Academic Model Providing Access to Healthcare (AMPATH) consortium. The AMPATH HIV care program represents a 20-year partnership among Indiana University School of Medicine (IUSM), Moi University School of Medicine (MUSM), and the Moi Teaching and Referral Hospital (MTRH) in Eldoret, Kenya. AMPATH has enrolled over 200,000 patients and currently provides care for approximately 15,000 children who are HEU and HIV+ in 65 clinics in western Kenya (9, 10). The children and their caregivers were recruited from the MTRH and AMPATH pediatric HIV clinics between 12/2017 and 9/2019, where care for prevention of mother-to-child transmission (PMTCT) of HIV has been freely provided since 2003 (11). Mixed convenience sampling of potential participants occurred within these clinics, with periods of consecutive sampling by study team members positioned within the clinic along with the clinic staff notifying the study team of eligible participants when the team was not physically present. Inclusion criteria included: (1) child between 18 and 36 months of age; (2) Kiswahili or English are the primary languages; (3) attending the MTRH maternal-child health (MCH) clinic or AMPATH HIV clinics; and (4) primary caregiver ≥18 years of age. Children aged 18–36 months are the population of interest in this study for the following reasons: (1) The young age ( ≤3 years) promotes early referral to intervention services during a critical period of child neurodevelopment, when intervention is most cost- and time-effective; (2) With 18 months as the lower age limit, cognitive and language domains may be tested with greater rigor and persisting delays will be more perceptible, while still allowing adequate time for intervention during this critical period; (3) From a feasibility standpoint, 18–36 months is the upper age limit of children born to mothers living with HIV routinely attending the MCH clinics in Kenya, due to the timing of their final HIV testing. We aimed to recruit 75 participants from each group (HEU, HIV+, and HUU), with HIV status obtained from maternal report. In total, 187 children were recruited. Twelve children could not complete ≥1 sub-sections of the Bayley-3, despite an additional time allowed to rest; two were excluded due to over-recruitment within the HEU cohort; and one did not return after consenting, resulting in 172 remaining study participants.

Perinatal data were not collected from participants. However, standard PMTCT guidelines at this time included an efavirenz-based first-line ART for adults living with HIV ≥15 years of age (12, 13).

### Bayley Scales of Infant and Toddler Development, 3rd Edition (Bayley-3)

The Bayley Scales of Infant and Toddler Development, 3rd edition (Bayley-3) is a neurodevelopment assessment that is internationally known and commonly used in research settings (1, 14). Our research team conducted a psychometric analysis of this culturally-adapted Bayley-3 within this setting in Kenya and found it to be valid and internally reliable (15). The cognitive, language (receptive/expressive), and motor (fine/gross) domains of the Bayley-3 were selected for use within this study, as they were the domains which had been culturally adapted. The Social-Emotional and Adaptive Behavior domains were not adapted due to the cross-cultural challenges in interpreting appropriate emotional status and behavior when compared to the normative population. One research assistant received training and approval to administer the Bayley-3 by a certified trainer and was blinded to study participants' HIV status prior to Bayley-3 administration.

### Data Collection

Caregivers completed a questionnaire containing demographic information and basic medical history, including birth history and HIV status. While clinical staff aided in identifying potentially eligible study participants, the HIV status was self-reported by caregivers. The caregiver also provided information on depressive symptoms using the Patient Health Questionnaire (PHQ-9) (16), UNICEF multiple cluster survey questions related to child stimulation (17), and socioeconomic status (SES), measured using the Wealth-Assets-Maternal Education-Income (WAMI index) (18). Anthropometric measurements were obtained at study staff. Each child's body mass and standing height was measured twice and if a discrepancy existed between the two measurements, the average was taken. Standing height was obtained in children <24 months to optimize measurement precision within the cohort. Weight was recorded to 0.1 kg and height was recorded to the 5 mm. Scale used: Salter 9,028 Razor Ultra Slim Technology Electronic Scale. Height measured with Seca 213 Portable Stadiometer Height-Rod. A blood sample was taken at enrollment to measure iron-deficiency anemia (IDA) using hemoglobin and serum ferritin. While the primary data regarding IDA is presented elsewhere (19), this variable was included within the regression analysis, as it is a known risk factor for worse neurodevelopmental outcomes (20). We classified a child as having IDA if (1) hemoglobin concentration was <118 g/L, based on World Health Organization-published algorithm for individuals living ≥2,000 meters in elevation, consist with local elevation (16); and (2) ferritin concentrations <12 μg/L. Study data were managed using REDCap, hosted at IUSM (21).

### Sample Size Calculation

At the time of this study's inception, data were limited regarding Bayley-3 scores within an international setting with children who were HEU and HUU. We used differences in rates of developmental delay among children who were HIV+ and HEU (22) to perform sample size calculations. With a margin of error of 5 points and 95% confidence interval, a sample size of 72 individuals per group was needed to determine a difference among groups. We aimed to recruit 75 per group to allow for a number who may not complete all study activities.

### Statistical Analysis

Characteristics of children and caregivers were summarized for the total cohort and by HIV status. For continuous variables, the mean and standard deviation (SD) were reported, and differences among HIV groups were assessed using one-way ANOVA. For categorical variables, the frequency and percentage were presented, and proportions were compared using the chi-square test. Anthropometric measurements (height and weight) were converted to height-for-age z-scores, weight-for-age z-scores, and weight-for-height z-scores using the modeling defined by the WHO (23). Multivariable linear regression models were used to examine the associations between scores from neurodevelopmental subtests and predictors. Potential predictors were identified using two methods: (1) *a priori* using clinical judgement and (2) when corresponding *p*-values of 0.05 were found among the three groups (HIV+, HEU, and HUU) within univariable analyses. Main effect estimates, standard errors (SE), 95% confidence intervals (CI), and significance tests for the predictors were produced.

### Ethics

All caregivers provided written informed consent for their child's participation in this study. The study was approved by Institutional Review Boards at IUSM in Indianapolis, USA and MUSM in Eldoret, Kenya.

## Results

Of the 187 children enrolled, 172 (*n* = 24 HIV+; *n* = 74 HEU; *n* = 74 HUU) had Bayley-3 data and were included in the analysis. The mean age was 23.1 (SD: 4.6) months. Children who were HIV+ were older compared to children who were HEU and HUU (26.0 vs. 21.7 and 23.5, respectively; *p* < 0.001). While not part of the inclusion criteria, all caregivers were mothers. Mothers of children who were HEU had significantly higher levels of depressive symptoms (*p* = 0.002) compared to their HIV+ or HUU peers (Table 1). Children who were HUU had mothers with higher levels of education (*p* < 0.001) and household resources (*p* < 0.001) compared to children who were HEU or HIV+.

**Table 1:** Characteristics of study population, recruited from a large referral hospital in western Kenya, at 18–36 months.

	**Overall (*N* = 172)**	**HIV-infected (*n* = 24)**	**HIV-exposed (*n* = 74)**	**HIV-unexposed (*n* = 74)**	***P* value**
**Children, mean (SD)**					
Age, in months	23.1 (4.6)	26.0 (5.5)	21.7 (3.6)	23.5 (4.6)	**<0.001**
Weight, in kg	11.5 (1.8)	12.4 (1.9)	11.0 (1.7)	11.6 (1.8)	**0.003**
Height, in cm	82.3 (6.2)	85.5 (6.9)	80.7 (5.6)	82.9 (6.0)	**0.003**
**Gender**, ***n*** **(%)**					
Female	83 (48.3)	11 (45.8)	39 (52.7)	33 (44.6)	0.595
Male	89 (51.7)	13 (54.2)	35 (47.3)	41 (55.4)	
**Preterm (<37 weeks)**, ***n*** **(%)**					
Yes	13 (7.8)	1 (4.6)	9 (12.7)	3 (4.1)	0.127
No	154 (92.2)	21 (95.5)	62 (87.3)	71 (96.0)	
**Mothers**, ***n*** **(%)**					
**Maternal education**					
No school/partial or complete primary school	61 (35.5)	9 (37.5)	40 (54.1)	12 (16.2)	**<0.001**
Some or complete secondary school/post-secondary school	111 (64.5)	15 (62.5)	34 (46.0)	62 (83.8)	
Maternal concerns for developmental delay in child, Yes	31 (18.0)	3 (12.5)	14 (18.9)	14 (18.9)	0.750
Community concern for developmental delay in child, Yes	18 (10.5)	3 (12.5)	9 (12.2)	6 (8.1)	0.680
Family history of developmental delays, Yes	23 (13.5)	0	14 (18.9)	9 (12.3)	0.063
Children's books in the home (any with >0), Yes	38 (22.1)	3 (12.5)	16 (21.6)	19 (25.7)	0.398
Left completely alone at least 1 h in the past week, Yes	26 (15.1)	5 (20.8)	11 (14.9)	10 (13.5)	0.683
Left with an older child at least 1 h in the past week, Yes	65 (38.0)	8 (33.3)	31 (42.0)	26 (35.6)	0.646
PHQ-9, Mean (SD)	3.5 (4.0)	2.6 (2.9)	4.7 (4.2)	2.5 (3.7)	**0.002**
WAMI, Mean (SD)	0.65 (0.20)	0.59 (0.21)	0.58 (0.20)	0.73 (0.18)	**<0.001**

*CM, centimeter; KG, kilogram; PHQ-9, Patient Health Questionnaire; SD, standard deviation; WAMI, Water-Assets-Maternal Education Index. Bolding indicates these values met statistical significance, with alpha set at 0.05*.

No statically significant differences were found among the three groups in cognition, expressive language, fine motor, or gross motor domains, with the exception that children who were HIV+ were found to have higher receptive language scores than others (*p* = 0.007). Generally, children who were HEU had lower scores than children who were HIV+ or HUU (Table 2).

**Table 2:** Developmental scores and anthropometrics of study population, aged 18–36 months, by HIV status.

	**Overall (*N* = 172)**	**HIV-infected (*n* = 24)**	**HIV-exposed (*n* = 74)**	**HIV-unexposed (*n* = 74)**	***P*** **-value**
**Developmental scores – Bayley-3, mean (SD)**					
**Composite score**					
Cognitive	74.4 (11.3)	74.0 (10.5)	73.7 (11.5)	75.1 (11.5)	0.737
Language	75.5 (13.8)	79.7 (16.8)	73.4 (13.7)	76.3 (12.7)	0.125
Motor	81.7 (11.4)	82.9 (11.8)	81.6 (12.5)	81.3 (10.3)	0.846
**Scaled score**					
Cognitive	4.9 (2.3)	4.8 (2.1)	4.7 (2.3)	5.0 (2.3)	0.737
Receptive language	5.2 (2.1)	6.3 (2.4)	4.8 (1.9)	5.2 (2.0)	**0.008**
Expressive language	6.4 (3.2)	6.7 (3.9)	6.1 (3.1)	6.7 (3.0)	0.442
Fine motor	8.4 (2.5)	9.0 (2.2)	8.6 (2.7)	8.1 (2.5)	0.240
Gross motor	5.5 (1.9)	5.3 (2.5)	5.4 (1.9)	5.7 (1.8)	0.486
**Anthropometrics z-scores, mean (SD)**					
Weight-for-age	−0.2 (1.1)	0.00 (0.9)	−0.3 (1.1)	−0.2 (1.1)	0.355
Height-for-age	−1.2 (1.5)	−0.9 (1.7)	−1.4 (1.4)	−1.1 (1.4)	0.283
Weight-for-height	0.5 (1.0)	0.4 (1.3)	0.5 (1.0)	0.5 (0.9)	0.876

*Bayley-3, Bayley Scales of Infant and Toddler Development; 3rd edition; SD, Standard Deviation. Bolding indicates these values met statistical significance, with alpha set at 0.05*.

When performing adjusted regression analyses, lower height-for-age z-scores were associated with lower scores in expressive language, fine motor, and gross motor (Table 3). Being left at home alone for at least 1 h within the past week was associated with lower scores in receptive language and expressive language. Higher SES scores were associated with lower fine and gross motor scores.

**Table 3:** Adjusted linear regression analysis of study population, aged 18–36 months, by Bayley-3 subtest.

**Variable**	**Comparison**	**Cognitive scaled score**		**Receptive language scaled score**		**Expressive language scaled score**		**Fine motor scaled score**		**Gross motor scaled score**	
		**Estimate (SE)**	**95%CI**	**Estimate (SE)**	**95%CI**	**Estimate (SE)**	**95%CI**	**Estimate (SE)**	**95%CI**	**Estimate (SE)**	**95%CI**
Age in months	One-month increase	−0.05 (0.05)	(−0.15 to 0.05)	**0.09 (0.04)**	**(0.01, 0.17)**	−0.06 (0.06)	(−0.18 to 0.07)	−0.06 (0.05)	(−0.16 to 0.04)	0.004 (0.04)	(−0.08 to 0.09)
Gender	Female vs. Male	0.60 (0.49)	(−0.38 to 1.58)	0.32 (0.41)	(−0.49 to 1.13)	−0.07 (0.63)	(−1.33 to 1.18)	**1.15 (0.51)**	**(0.14, 2.15)**	0.29 (0.41)	(−0.53 to 1.10)
Preterm <37 weeks	Yes vs. No	1.06 (0.86)	(−0.64 to 2.76)	−0.42 (0.71)	(−1.83 to 0.99)	0.72 (1.09)	(−1.45 to 2.89)	0.38 (0.88)	(−1.37 to 2.12)	0.16 (0.71)	(−1.25 to 1.57)
Maternal concerns for developmental delay	Yes vs. No	0.06 (0.69)	(−1.32 to 1.44)	−0.99 (0.58)	(−2.14 to 0.15)	**−2.04 (0.89)**	**(−3.80 to** **−0.28)**	−0.64 (0.71)	(−2.05 to 0.77)	−0.15 (0.58)	(−1.29 to 0.99)
Maternal depression, by PHQ−9	One-unit increase	−0.04 (0.06)	(−0.17 to 0.08)	0.04 (0.05)	(−0.06 to 0.15)	0.004 (0.08)	(−0.16 to 0.17)	−0.03 (0.07)	(−0.16 to 0.10)	−0.10 (0.05)	(−0.20 to 0.01)
WAMI	One-standard deviation increase	−0.16 (0.29)	(−0.72 to 0.41)	−0.24 (0.24)	(−0.24 to 0.23)	−0.59 (0.36)	(−1.31 to 0.13)	**−0.82 (0.29)**	**(−1.40 to** **−0.24)**	**−0.48 (0.24)**	**(−0.95 to** **−0.01)**
Height-for-age z-score	One-unit decrease	−0.17 (0.16)	(−0.14 to 0.49)	−0.07 (0.13)	(−0.19 to 0.33)	**−0.43 (0.20)**	**(−0.02 to** **−0.83)**	**−0.40 (0.16)**	**(−0.07 to** **−0.72)**	**−0.37 (0.13)**	**(−0.11 to** **−0.63)**
Children's books in the home	Yes vs. No	−0.71 (0.61)	(−1.92 to 0.49)	0.69 (0.50)	(−0.30, 1.69)	0.10 (0.77)	(−1.44, 1.64)	0.61 (0.62)	(−0.62, 1.84)	0.27(0.50)	(−0.73, 1.26)
Left alone at least 1day in the past week	Yes vs. No	−0.41 (0.64)	(−1.68 to 0.87)	**−1.08 (0.53)**	**(−2.14 to** **−0.03)**	**−2.07 (0.82)**	**(−3.70 to** **−0.44)**	−0.92 (0.66)	(−2.23 to 0.39)	−0.83 (0.53)	(−1.89 to 0.23)
HIV status	HIV-exposed, uninfected vs. HIV-unexposed	−0.23 (0.54)	(−1.30 to 0.83)	−0.32 (0.44)	(−1.20 to 0.56)	−1.20 (0.69)	(−2.56 to 0.16)	−0.08 (0.55)	(−1.17 to 1.01)	−0.43 (0.44)	(−1.31 to 0.45)
	HIV-infected vs. HIV-unexposed	0.35 (0.80)	(−1.24 to 1.93)	0.78 (0.66)	(−0.54 to 2.09)	0.25 (1.02)	(−1.78 to 2.28)	0.44 (0.82)	(−1.19 to 2.06)	−0.90 (0.66)	(−2.21 to 0.42)
Iron Deficiency Anemia	Yes vs. No	0.75 (0.53)	(−0.31 to 1.80)	−0.003 (0.44)	(−0.88 to 0.87)	0.41 (0.68)	(−0.94 to 1.76)	−0.59 (0.55)	(−1.67 to 0.49)	−0.45 (0.44)	(−1.33 to 0.42)

*Bayley-3, Bayley Scales of Infant and Toddler Development, 3rd edition; CI, Confidence Interval; PHQ-9, Patient Health Questionnaire; SE, Standard Error; WAMI, Water-Assets-Maternal Education Index. Bolding indicates these values met statistical significance, with alpha set at 0.05*.

## Discussion

This small cross-sectional pilot study did not show consistent neurodevelopmental differences among children who were HIV+, HEU, and HUU, although children who were HIV+ did have higher receptive language scores. Being stunted, left completely alone for at least an hour within the last week, and having higher SES were associated with worse neurodevelopmental scores. Mothers of children who were HEU reported more symptoms of depression than other mothers. This pilot study provides useful insights on additional variables needed within a larger scale neurodevelopmental study in populations of children who are HEU.

Stunting is a well-known risk factor for poor neurodevelopment (24) and often used as a proxy measure for at-risk development in the absence of more specific data (25). Malnutrition risk is also higher in populations with HIV infection and exposure (26–28). Stunting was associated with worse neurodevelopmental scores within our study cohort, but HIV status was not. In fact, while not statistically significant, children who were HIV+ had the highest mean anthropometric z-scores among the three groups and also had higher scores on receptive language. Due to the small sample of children who are HIV+, these results should be interpreted with caution. However, one may hypothesize that children who are HIV+ at AMPATH were more engaged in care compared to others due to HIV surveillance. This enhanced engagement may have allowed for earlier detection of growth problems and referrals to food programs (29). It is also possible that families may be more concerned for the health of their children who are HIV+ and provide them additional food. We did not collect data on participation in support programs or food availability, which may have differed between groups. Future studies should obtain this data to determine the potentially confounding effect of food supports on neurodevelopment outcomes of populations affected by HIV.

Mothers of children who were HEU expressed significantly more depressive symptoms than those of children who were HIV+ and HUU. While this was not associated with worse neurodevelopmental outcomes in our cohort, maternal depression is a known risk factor for worse development in other populations (30, 31). The lack of information on supports utilized by families affected by HIV limits our understanding of this finding. However, it highlights the issue that siloed care and support systems helping one specific population may unintentionally leave others behind. Any mother living with HIV+ may experience HIV stigma or job discrimination, but in some settings, only those with children who are HIV+ may qualify to access additional resources for their children. More information on the effects of siloed HIV care and depression in mothers of children who are HEU is needed.

In sub-Saharan Africa, an estimated 35% of children <5 years are left at home alone or with a young sibling for >1 h/week (32). Within our study, nearly 15% of children were left completely alone for ≥1 h within the prior week, which was associated with lower receptive and expressive language scores. Poverty, lack of societal support, and local cultural norms are drivers for parental decisions to leave young children home alone (33). While data clearly links lack of supervision with unintentional injury in young children (34), its impact on development in young children has not been well-studied. For school-aged children, being left home alone ≥1 day/week increases conduct problems, hyperactivity and intention symptoms, and peer relationship problems compared to well-supervised peers (35, 36). More research is needed to inform policies and interventions to support families in creating safe, nurturing environments for their children (37).

We found that children with higher SES had lower motor scores. Fine and gross motor scores may be less sensitive to socioeconomic differences (38, 39); however, poverty and lower SES are generally strong risk factors for worse development (24, 40). Within our setting, a few hypotheses may be considered for this finding. Firstly, households with working mothers likely have higher SES, but often necessitate household workers who may provide suboptimal quality childcare. Further, within this context, televisions are more commonly found in households with high SES, and it may be possible that these young children were exposed to substantial television time, which is associated with increased rates of cognitive, language, and motor delays (41). A more comprehensive evaluation of potentially confounding variables, including social supports and household resources, would be helpful in exploring additional hypotheses for this finding in the future.

Our small sample size limited our power to show a difference in neurodevelopment among our three groups, with a particularly small cohort of children who are HIV+. This was a primary limitation of this study. While HIV prevalence among Kenyan women of reproductive age is 6.1% (42), various policy changes and expansion of services have resulted in significant reductions in vertical transmission in Kenya, from 26% in 2009 to 11% in 2018 (13, 43–46). The low numbers of recruited children who are HIV+ is a testament to successful PMTCT in Kenya and within the AMPATH program. However, the high-quality care the children who were HIV+ received through this program may have biased the results toward the null within this study. Furthermore, survival bias may have influenced the number of children who are HIV+ who were recruited to this study and their outcomes, higher anthropometrics and fewer pre-term births, as enrolment did not occur until 18 months of age. Additionally, the inclusion of children who were HUU attending MCH clinic at 18–36 months may also have biased the results toward the null, as many healthy children stop attending the MCH after their 9 month immunizations despite recommendations to attend until 5 years of age (47). Those children who continue coming to the clinic may have more health conscientious caregivers, or perhaps more likely, may have ongoing health issues that require follow up. This is a limitation of this study inherent to its cross-sectional nature and convenience sampling. This pilot study was also limited by the lack of information on *in-utero* environment, maternal medications, and additional supports offered to families. Also, HIV status was primarily self-reported by mothers, which may have led to mis-classification. Our study team performed careful questioning around HIV status, which ultimately resulted in over recruitment in children who were HEU. During the study visit, two children whose mothers initially self-reported as being HUU were found to be HEU upon further questioning. Further, we did not ask mothers who the primary caregiver was during the day, as house help or family members often provide care while mothers are working. It is critically important that funding of research accommodates prospective data collection, from the period of earliest fetal exposure, namely conception, throughout the period of interest for the child, to ensure that rigorous research is conducted among children who are HEU. Despite these challenges, our study provided high quality data using an adapted Bayley-3.

Of note, the scaled scores within this study were much lower than the normative data for the Bayley-3, with our mean scaled sub-test scores falling >1 SD below the normed scaled mean. Reliance on Bayley-3's U.S. population reference curves has been shown to misclassify children in other cultures (48). In a sample of young Malawian children, raw Bayley-3 scores from the normative data and the Malawian sample were similar until children reached 9–12 months of age, after which the Malawian children's raw scores increased at a slower pace compared to the normative sample (48). A few potential hypotheses are posed for why this may occur. It is possible that the non-U.S. children may not have optimal environments for development. Only a third of mothers within this study completed education beyond secondary school, which is known to be a strong predictor of lower developmental scores (49). Additionally, some necessary alterations made to the test administration during the cultural adaptation process may have negatively impacted scoring when compared to the standard administration (15). A third hypothesis is that certain aspects of development are more adaptive and highly prioritized in settings like Kenya compared to the U.S. but are not measured with assessments like the Bayley-3, thereby resulting in a lower score. As a general example of expectation differences between cultures, we previously found that Kenyan clinical providers were concerned when children were not walking independent by 12 months of age (50), whereas, the U.S. Centers for Disease Control expects that most children will not walk independently until 18 months (51). As with all assessments, the items are evaluated are on the constructs deemed important by the test developers, which may introduce scoring bias. Thus, these results are only to be used to compare scores among our three study groups and not to be directly compared to other populations.

## Conclusions

Within our study population, being stunted and left at home alone were associated with worse neurodevelopment. Additional research is needed to explore neurodevelopment in children impacted by HIV, especially the growing population of children who are HEU. A more complete participant history with comprehensive documentation of potentially confounding variables, such as nutritional and household support and ARV exposure, is necessary to determine the true effects of HIV exposure or infection on neurodevelopmental outcomes. Screening mechanisms are needed to detect early risk for worse health and neurodevelopmental outcomes in child who are HEU, so that targeted interventions may be introduced to optimize long-term outcomes.

## Data Availability Statement

The raw data supporting the conclusions of this article will be made available by the authors, without undue reservation.

## Ethics Statement

The studies involving human participants were reviewed and approved by Institutional Review Boards at Indiana University School of Medicine in Indianapolis, USA and Moi University School of Medicine in Eldoret, Kenya. Written informed consent to participate in this study was provided by the participants' legal guardian/next of kin.

## Author Contributions

MM, EO, and RV designed the research study during MSM's training program. AO and CC performed the research and provided insights for the findings, with supervision by EO, RV, and MM. ZY and MM analyzed the data. MM wrote the paper, with input of all others. All authors contributed to the article and approved the submitted version.

## Funding

This study was funded by Indiana University's Morris Green Physician Scientist Development Program and Indiana CTSI at Indiana University School of Medicine. MSM's time during the implementation of this study was funded by a training grant entitled Training in STIs and Other Infections of Global Health Significance (T32AI007637, PI: Dr. Wools Kaloustian) from the National Institute of Allergy and Infectious Diseases, Bethesda, MD, USA.

## Conflict of Interest

The authors declare that the research was conducted in the absence of any commercial or financial relationships that could be construed as a potential conflict of interest.

## Publisher's Note

All claims expressed in this article are solely those of the authors and do not necessarily represent those of their affiliated organizations, or those of the publisher, the editors and the reviewers. Any product that may be evaluated in this article, or claim that may be made by its manufacturer, is not guaranteed or endorsed by the publisher.
